# Large increase in bloodstream infections with carbapenem-resistant *Acinetobacter* species during the first 2 years of the COVID-19 pandemic, EU/EEA, 2020 and 2021

**DOI:** 10.2807/1560-7917.ES.2022.27.46.2200845

**Published:** 2022-11-17

**Authors:** Pete Kinross, Carlo Gagliotti, Hanna Merk, Diamantis Plachouras, Dominique L Monnet, Liselotte Diaz Högberg, EARS-Net Study Group, EARS-Net Study Group participants, Reinhild Strauss, Karl Mertens, Stefana Sabtcheva, Arjana Tambic Andrasevic, Panagiota Maikanti, Helena Žemličková, Henrik Hasman, Marina Ivanova, Kati Räisänen, Sylvie Maugat, Ines Noll, Kassiani Mellou, Ákos Tóth, Kristján Orri Helgason, Stephen Murchan, Giulia Errico, Ieva Voita, Esther Walser-Domjan, Jolanta Miciulevičienė, Monique Perrin, Elizabeth Anne Scicluna, Sjoukje HS Woudt, Ørjan Samuelsen, Dorota Żabicka, Manuela Caniça, Gabriel Adrian Popescu, Eva Schréterová, Helena Ribič, Maria Belén Aracil García, Hanna Billström

**Affiliations:** 1 European Centre for Disease Prevention and Control (ECDC), Stockholm, Sweden; 2 Regional Agency for Health and Social Care of Emilia-Romagna, Bologna, Italy; 3 Members of the EARS-Net Study Group are listed under Collaborators and at the end of the article; * These authors contributed equally to the article and share first authorship.

**Keywords:** Acinetobacter, bacteraemia, EU/EEA, COVID-19, pandemic

## Abstract

Recent data from the European Antimicrobial Resistance Surveillance Network (EARS-Net) show a large increase of +57% in *Acinetobacter* species bloodstream infections in the European Union and European Economic Area in the first years of the COVID-19 pandemic (2020–2021) compared with 2018–2019. Most were resistant to carbapenems, from intensive care units, and in countries with ≥ 50% carbapenem resistance in *Acinetobacter* spp. in 2018–2019. This highlights the requirement for reinforced *Acinetobacter* preparedness and infection prevention and control in Europe.

Bloodstream infections (BSIs) with *Acinetobacter* species commonly have poor outcomes, especially in intensive care unit (ICU) patients [1]. *Acinetobacter* spp. is intrinsically resistant to many antimicrobials, and additional acquired resistance further complicates the treatment of serious infections in already vulnerable patient groups. Recent data from the European Antimicrobial Resistance Surveillance Network (EARS-Net) show a large and statistically significant increase in reports of *Acinetobacter* spp. BSIs in the European Union (EU) and European Economic Area (EEA) during the period from 2017 to 2021 [2]. Most of this increase occurred in 2020 and 2021, the first years of the coronavirus disease (COVID-19) pandemic. Here we further explore this trend in a subset of data from laboratories that continuously reported data during that period.

## Data description

Our data originate from qualitative routine antimicrobial susceptibility testing (AST) results of blood isolates collected by local clinical laboratories in national networks in EU/EEA countries. These results are reported annually by national centres to the European Centre for Disease Prevention and Control (ECDC), according to the EARS-Net reporting protocol [3]. In its analyses, EARS-Net only includes the first isolate per patient each year and for each bacterial species.

All EU countries, Iceland and Norway reported data to EARS-Net every year during the period 2017 to 2021 [2,4]. For this analysis, we restricted the dataset to BSIs with *Acinetobacter* spp. and to only those laboratories that reported carbapenem (imipenem and/or meropenem) antimicrobial susceptibility testing results for *Acinetobacter* spp. for every year in 2017 to 2021 (255 of 826 laboratories reporting, on average, per year). We made this restriction to limit bias from year-to-year changes in the number, hospital affiliation and type of reporting laboratories, and because not all countries can discriminate between laboratories that did not report and those that had no cases. The United Kingdom ceased reporting data to ECDC in 2020 when it withdrew from the EU and was hence not included. In addition, France was excluded because, following a major reorganisation of national surveillance in 2020, only a few laboratories were continuously identifiable. The Table presents data for the 28 included countries, with and without restriction to continuously reporting laboratories.

**Table:** Annual carbapenem-resistant *Acinetobacter* species bloodstream infections and carbapenem susceptibility testing results for *Acinetobacter* species bloodstream infections, in all laboratories that reported data to EARS-Net and in those that continuously reported data, EU/EEA countries, 2017–2021 (n = 31,242 infections)

Carbapenem susceptibility test result	2017		2018		2019		2020		2021		Number of cases in 2020–2021 vs 2018–2019		Number of cases in 2020 vs average 2017–2019		Number of cases in 2021 vs average 2017–2019	
	n	%	n	%	n	%	n	%	n	%	% Change	p value^c^	% Change	p value^c^	% Change	p value^c^
All laboratories (annual mean = 826 laboratories)^a^																
R	2,831	59.2	3,105	60.1	2,629	56.5	4,379	65.2	7,396	74.5	+105.4	<0.001^d^	+53.4	<0.001^d^	+159.1	<0.001^d^
S/I	1,950	40.8	2,063	39.9	2,028	43.5	2,333	34.8	2,528	25.5	+18.8	<0.001	+15.9	<0.001	+25.5	<0.001
All	4,781	100.0	5,168	100.0	4,657	100.0	6,712	100.0	9,924	100.0	+69.3	<0.001	+37.9	<0.001	+103.8	<0.001
Continuously reporting laboratories (n = 255 laboratories)^a,b^																
R	1,237	48.5	1,293	48.3	1,354	48.4	1,891	57.6	3,767	70.8	+113.8	<0.001^d^	+46.1	<0.001^d^	+191.0	<0.001^d^
*R (Group 1 countries^e^)*	*30*	3.6	*37*	4.4	*17*	1.9	*29*	3.3	*23*	2.3	*−3.7*	*0.85*	*+3.6*	*0.90*	*−17.9*	*0.49*
*R (Group 2 countries^e^)*	*60*	35.3	*38*	21.5	*26*	20.6	*30*	22.7	*104*	49.8	*+109.4*	*<0.001^d^*	*−27.4*	*0.18*	*+151,6*	*<0.001^d^*
*R (Group 3 countries^e^)*	*1,147*	74.6	*1,218*	73.5	*1,311*	72.9	*1,832*	80.6	*3,640*	88.3	*+116.4*	*<0.001^d^*	*+49.5*	*<0.001^d^*	*+197.1*	*<0.001^d^*
S/I	1,314	51.5	1,382	51.7	1,444	51.6	1,390	42.4	1,554	29.2	+4.2	0.12	+0.7	0.85	+12.6	0.001
S/I/R	2,551	100.0	2,675	100.0	2,798	100.0	3,281	100.0	5,321	100.0	+57.2	<0.001	+22.7	<0.001	+98.9	<0.001

EARS-Net: European Antimicrobial Resistance Surveillance Network; EEA: European Economic Area; EU: European Union; I: susceptible, increased exposure; R: resistant; S: susceptible, standard dosing regimen.

^a^ Data for France and the United Kingdom were excluded.

^b^ Only includes EU/EEA laboratories that were identifiable as having reported ≥ 1 *Acinetobacter* spp. isolate with carbapenem susceptibility data every year in 2017–2021.

^c^ Poisson regression model to assess the statistical significance of changes in the numbers of bloodstream infections.

^d^ The chi-squared test comparing the percentage of carbapenem resistance in 2020–2021 vs 2018–2019 also had p < 0.001.

^e^ The reporting countries were grouped according to the mean of their crude, national, annual percentage of *Acinetobacter* spp. resistance to carbapenems in 2018 and 2019. These were Group 1 (< 10% carbapenem resistance in 2018–2019): Austria, Belgium, Denmark, Estonia, Finland, Germany, Iceland, Ireland, Luxembourg, Malta, the Netherlands, Norway and Sweden; Group 2 (10% to < 50% carbapenem resistance in 2018–2019): Czechia, Portugal, and Slovenia; Group 3 (≥ 50% carbapenem resistance in 2018–2019): Bulgaria, Croatia, Cyprus, Greece, Hungary, Italy, Latvia, Lithuania, Poland, Romania, Slovakia and Spain. Percentages in these rows refer to carbapenem-resistant isolates among all isolates analysed for the countries in a given group. The raw data for the group level are provided in the Supplement.

As the resistance percentages for *Acinetobacter* spp. varied substantially between EU/EEA countries [4], we grouped the countries according to their mean national annual carbapenem resistance percentage in 2018—2019. Countries in Group 1 had < 10% carbapenem resistance (n = 13; Austria, Belgium, Denmark, Estonia, Finland, Germany, Iceland, Ireland, Luxembourg, Malta, the Netherlands, Norway, Sweden), Group 2 had 10% to < 50% carbapenem resistance (n = 3: Czechia, Portugal, Slovenia) and Group 3 had ≥ 50% carbapenem resistance (n = 12: Bulgaria, Croatia, Cyprus, Greece, Hungary, Italy, Latvia, Lithuania, Poland, Romania, Slovakia, Spain). When stratifying by patient ward type, we grouped the units as ‘ICU’ (adult and paediatric ICUs), ‘not ICU’ (all other ward types) and ‘unknown ward type’ (no information available on ward type).

We assessed the statistical significance of changes in the numbers of BSIs and in the percentage of carbapenem resistance comparing 2020–2021 with 2018–2019, using Stata Statistical Software (Release 15.1. College Station, TX: StataCorp LLC) for a Poisson regression model and a chi-squared test, respectively, with p values < 0.05 considered as significant.

## Trends in *Acinetobacter* species bloodstream infections from continuously reporting EU/EEA laboratories

**Figure 1:**
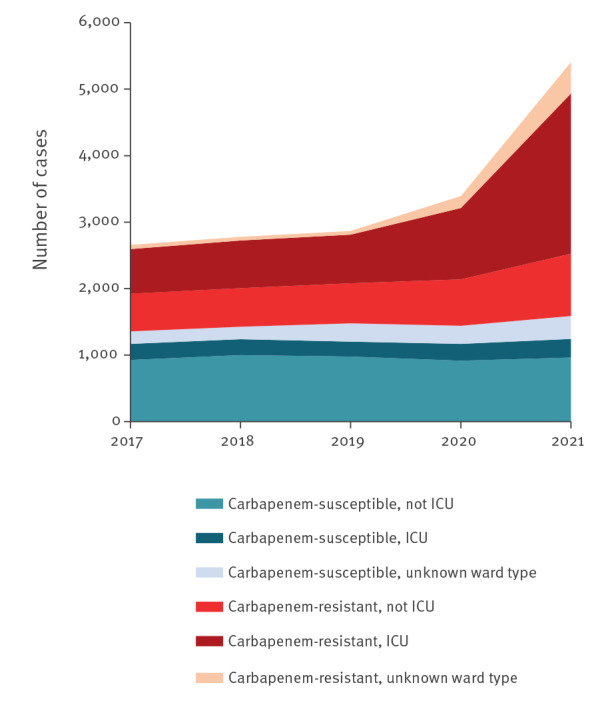
*Acinetobacter* species bloodstream infections reported by laboratories that continuously reported data to EARS-Net, by carbapenem susceptibility testing result and type of patient ward, EU/EEA, 2017–2021 (n = 16,626)

The total number of *Acinetobacter* spp. BSIs reported in 2020–2021 increased by + 57% compared with 2018–2019 (p < 0.001). Most of this increase was due to carbapenem-resistant *Acinetobacter* spp. BSIs, with the number of reports increasing by + 114% (p < 0.001), and the carbapenem resistance percentage increasing from 48.4% in 2018–2019 to 65.8% in 2020–2021 (p < 0.001) (Table). The number of carbapenem-resistant *Acinetobacter* spp. BSIs increased more among ICU patients (+ 144%) than non-ICU patients (+ 41%) (Figure 1). The small increase in the number of carbapenem-susceptible *Acinetobacter* spp. BSIs in 2020–2021 compared with 2018–2019 was not significant (p = 0.12).

EARS-Net: European Antimicrobial Resistance Surveillance Network; EEA: European Economic Area; EU: European Union; ICU: intensive care unit.

Cases labelled carbapenem-susceptible include I (susceptible, increased exposure) and S (susceptible, standard dosing regimen).

**Figure 2:**
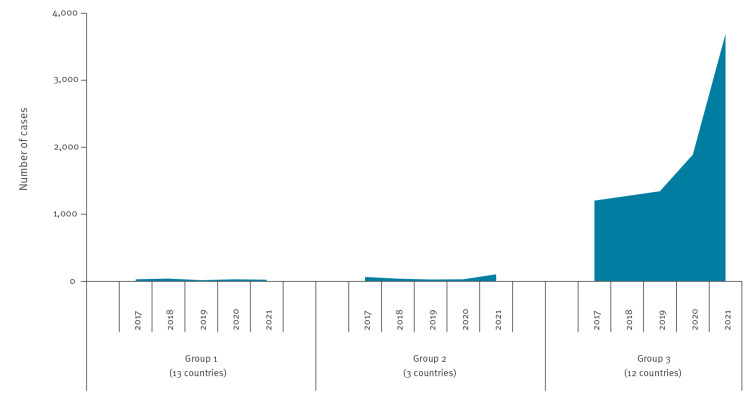
Bloodstream infections with carbapenem-resistant *Acinetobacter* species, reported by laboratories that continuously reported data to EARS-Net, by country group^a^ and year, EU/EEA, 2017–2021 (n = 9,542)

**Figure 3:**
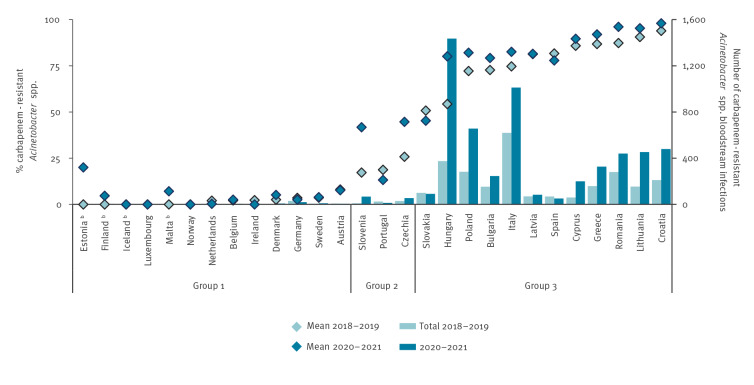
Percentage and number of bloodstream infections with carbapenem-resistant *Acinetobacter* species from laboratories that continuously reported data to EARS-Net, by country group^a^ , EU/EEA, 2018–2019 vs 2020–2021 (n = 9,542)

Countries in Group 3 (≥ 50% mean carbapenem resistance in *Acinetobacter* spp. in 2018–2019) experienced the most noticeable increases in the number of *Acinetobacter* spp. BSIs in 2020–2021. They had a statistically significant increase (p < 0.001) of + 116% in the number of reported cases in 2020–2021 (n = 5,472) compared with 2018–2019 (n = 2,529) (Table, Figure 2, Figure 3) [4]. In countries in Group 2, the increase was similar (+ 109%; p < 0.001), albeit with fewer reports per country (Table, Figure 3). Countries in Group 1 reported few cases (n = 52) in 2020–2021 and showed no significant change compared with 2018–2019 (n = 54; p = 0.85) (Table).

EARS-Net: European Antimicrobial Resistance Surveillance Network; EEA: European Economic Area; EU: European Union.

^a^ The reporting countries were grouped according to the mean of their crude, national, annual percentage of *Acinetobacter* spp. resistance to carbapenems in 2018 and 2019. These were Group 1 (< 10% carbapenem resistance in 2018–2019): Austria, Belgium, Denmark, Estonia, Finland, Germany, Iceland, Ireland, Luxembourg, Malta, the Netherlands, Norway and Sweden; Group 2 (10% to < 50% carbapenem resistance in 2018–2019): Czechia, Portugal, and Slovenia; Group 3 (≥ 50% carbapenem resistance in 2018–2019): Bulgaria, Croatia, Cyprus, Greece, Hungary, Italy, Latvia, Lithuania, Poland, Romania, Slovakia and Spain.

EARS-Net: European Antimicrobial Resistance Surveillance Network; EEA: European Economic Area; EU: European Union.

^a^ The reporting countries were grouped according to the mean of their crude, national, annual percentage of *Acinetobacter* spp. resistance to carbapenems in 2018 and 2019. These were Group 1 (< 10% carbapenem resistance in 2018–2019): Austria, Belgium, Denmark, Estonia, Finland, Germany, Iceland, Ireland, Luxembourg, Malta, the Netherlands, Norway and Sweden; Group 2 (10% to < 50% carbapenem resistance in 2018–2019): Czechia, Portugal, and Slovenia; Group 3 (≥ 50% carbapenem resistance in 2018–2019): Bulgaria, Croatia, Cyprus, Greece, Hungary, Italy, Latvia, Lithuania, Poland, Romania, Slovakia and Spain.

^b^ Reported < 25 *Acinetobacter* spp. isolates in both periods 2018–2019 and 2020–2021. As the data are restricted to laboratories that reported continuously in 2017–2021, the percentages and numbers for countries may differ from the dataset that included all laboratories (Table) [2,4].

For Latvia, the two datapoints for the mean overlap and only the dark symbol is visible.

## Discussion

The observed trends for *Acinetobacter* spp. BSI in the EU/EEA are worrying because resistance to carbapenems causes a high burden of disease in vulnerable hospitalised patients [5-7]. Our findings suggest that countries where carbapenem-resistant *Acinetobacter* spp. were already well established before the COVID-19 pandemic (Group 3) had the biggest challenges in controlling further spread in 2020–2021.

*Acinetobacter* spp. is difficult to eradicate from the hospital environment, colonising hospital patients and staff and causing outbreaks, particularly in ICUs [1]. Several reports have identified *Acinetobacter* spp. as one of the most frequent causes of infectious complications in hospitalised patients with COVID-19 [8-10]. The observed increasing trends at EU/EEA level compared with the pre-pandemic situation [2,11,12] were probably driven by the profound impact of the COVID-19 pandemic on hospital care, which increased the number of patients at risk of *Acinetobacter* spp. BSI, and also by difficulties in applying infection prevention and control (IPC) measures. In 2020–2021, there were larger numbers of severely ill patients, many with severe pulmonary infection. High occupancy rates necessitated increased provision of ICU beds, often with staff who were overworked or less experienced [13,14]. Inappropriate application of contact precautions for COVID-19 patients, in particular suboptimal hand hygiene, as well as contamination and insufficient cleaning of the hospital environment, probably contributed to direct or indirect between-patient *Acinetobacter* spp. transmission [15-20]. Finally, reduced attention to antimicrobial stewardship, with resulting increased carbapenem use, may have contributed [21].

For context, in 2020–2021 compared with 2018–2019, the laboratories that continuously reported *Acinetobacter* spp. data to EARS-Net also reported more cases of BSI with *Enterococcus faecium* (+ 29%), *E. faecalis* (+ 16%), *Pseudomonas aeruginosa* (+ 8%), *Klebsiella pneumoniae* (+ 6%), but these differences were much less pronounced than for BSI with *Acinetobacter* spp. (+ 57%). Laboratories reported fewer cases of BSI with *Streptococcus pneumoniae* (− 47%), *Escherichia coli* (− 5%) and *Staphylococcus aureus* (− 1%). These differences probably depend on the epidemiological characteristics of the various pathogens. For example, *S. pneumoniae* and *E. coli* are more frequently transmitted in the community and in non-ICU hospital settings. During the COVID-19 pandemic, transmission of microorganisms in the community was affected by containment actions such as stay-at-home orders, physical distancing, hygiene measures and the use of face masks. This may have contributed to the sharp decline in typically community-acquired infections such as those caused by *S. pneumoniae* [22,23].

There were exceptions to the general trends by country group, indicating that the trends were not only explained by the pre-pandemic percentage of carbapenem resistance. For example, Portugal and Spain were outliers in their respective groups by reporting fewer *Acinetobacter* spp. BSIs in 2020–2021 than in 2018–2019, whereas Slovenia reported a larger increase in *Acinetobacter* spp. BSIs than other Group 2 countries.

Although reasons for the trends observed during the COVID-19 pandemic remain to be clarified, most factors that potentially favoured the increase in carbapenem-resistant *Acinetobacter* spp. infections, and in general multidrug-resistant microorganisms, are amenable to public health intervention. Options include rigorous adherence to hand hygiene, environmental cleaning, provision and appropriate use of personal protective equipment, appropriate training of healthcare staff, and promotion of antimicrobial stewardship programmes. While spread of carbapenem-resistant *Acinetobacter* spp. is difficult to control while established, recent evidence shows that *Acinetobacter* spp. outbreaks can be controlled through a bundle of measures including thorough environmental cleaning, even without ward closure [16,24]. Finally, any country with an increasing number of infections with carbapenem-resistant *Acinetobacter* spp. in 2020–2021, particularly those with comparatively moderate resistance percentages (e.g. 10% to < 50%, Group 2), should urgently ensure preparedness for the prevention and control of *Acinetobacter* spp. infections and outbreaks.

## Conclusion

The large increase in carbapenem-resistant *Acinetobacter* spp. BSI in the EU/EEA during a time of great challenges for healthcare calls for reinforced application of the preparedness and response actions that we present above. Surveillance at local, national and EU/EEA levels will be vital to monitor whether this worrying development is halted or even reversed. 

## EARS-Net Study Group participants

Reinhild Strauss, Federal Ministry of Social Affairs, Health, Care and Consumer Protection, Vienna, Austria

Karl Mertens, Sciensano, Brussels; Belgium

Stefana Sabtcheva, National Oncology Centre - USHATO, Bulgaria

Arjana Tambic Andrasevic, University Hospital for Infectious Diseases, Zagreb, Croatia

Panagiota Maikanti, Nicosia General Hospital, Nicosia, Cyprus

Helena Žemličková, National Institute of Public Health, Prague, Czech Republic

Henrik Hasman, Referencelaboratoriet for antibiotikaresistens og Stafylokokker, Copenhagen, Denmark

Marina Ivanova, East-Tallinn Central Hospital Laboratory, Tallinn, Estonia

Kati Räisänen, Finnish Institute for Health and Welfare, Helsinki, Finland

Sylvie Maugat, Public Health France, Saint-Maurice, France

Ines Noll, Robert Koch Institute, Berlin, Germany

Kassiani Mellou, National Public Health Organization, Athens, Greece

Ákos Tóth, National Public Health Center, Hungary

Kristján Orri Helgason, Landspitali University Hospital of Iceland, Iceland

Stephen Murchan, Health Protection Surveillance Centre, Ireland

Giulia Errico, Istituto Superiore di Sanità, Italy

Ieva Voita, Centre for Disease Prevention and Control of Latvia, Riga, Latvia

Esther Walser-Domjan, Office of Public Health, Liechtenstein

Jolanta Miciulevičienė, National Public Health Surveillance Laboratory, Vilnius, Lithuania

Monique Perrin, Laboratoire national de Santé, Luxembourg

Elizabeth Anne Scicluna, Infection Control Unit, Mater Dei Hospital, Malta

Sjoukje H. S. Woudt, National Institute for Public Health and the Environment, the Netherlands

Ørjan Samuelsen, Norwegian National Advisory Unit on Detection of Antimicrobial Resistance; University Hospital of North Norway, Norway

Dorota Żabicka, National Medicines Institute, Poland

Manuela Caniça, National Institute of Health Dr. Ricardo Jorge, Portugal

Gabriel Adrian Popescu, Carol Davila University of Medicine and Pharmacy, Romania

Eva Schréterová, University Hospital of P.J.Šafárik Košice, Košice, Slovakia

Helena Ribič, National Institute of Public Health, Slovenia

Maria Belén Aracil García. Resistance Antibiotics Laboratory, National Center of Microbiology, Spain

Hanna Billström, The Public Health Agency of Sweden, Sweden

## Supplementary Data

109000 Supplement
