# Supplementary material for: Large increase in bloodstream infections with carbapenem-resistant Acinetobacter species during the first 2 years of the COVID-19 pandemic, EU/EEA, 2020 and 2021
Source: Euro Surveill. 2022 Nov 17;27(46):2200845. doi: 10.2807/1560-7917.ES.2022.27.46.2200845 (PMC9673235; doi:10.2807/1560-7917.ES.2022.27.46.2200845)
Supplement: Supplement [file 22-00845_KINROSS_Supplement.pdf]

This supplementary material is hosted by *Eurosurveillance* as supporting information alongside the article [Large increase in bloodstream infections with carbapenem-resistant *Acinetobacter* species during the first 2 years of the COVID-19 pandemic, EU/EEA, 2020 and 2021], on behalf of the authors, who remain responsible for the accuracy and appropriateness of the content. The same standards for ethics, copyright, attributions and permissions as for the article apply. Supplements are not edited by *Eurosurveillance* and the journal is not responsible for the maintenance of any links or email addresses provided therein.

Supplementary Table S1: Carbapenem-resistant and total *Acinetobacter* species bloodstream infections in laboratories that continuously reported data to EARS-Net, EU/EEA countries, 2017–2021

	2017			2018			2019			2020			2021		
	R	Total	%	R	Total	%	R	Total	%	R	Total	%	R	Total	%
Group 1	30	844	3.6	37	840	4.4	17	874	1.9	29	875	3.3	23	992	2.3
Group 2	60	170	35.3	38	177	21.5	26	126	20.6	30	132	22.7	104	209	49.8
Group 3	1,147	1537	74.6	1,218	1,658	73.5	1,311	1,798	72.9	1,832	2,274	80.6	3,640	4,120	88.3

EARS-Net: European Antimicrobial Resistance Surveillance Network; EEA: European Economic Area; EU: European Union; R: resistant.

The reporting countries were grouped according to the mean of their crude, national, annual percentage of *Acinetobacter* spp. resistance to carbapenems in 2018 and 2019. These were Group 1 (< 10% carbapenem resistance in 2018–2019): Austria, Belgium, Denmark, Estonia, Finland, Germany, Iceland, Ireland, Luxembourg, Malta, the Netherlands, Norway and Sweden; Group 2 (10% to < 50% carbapenem resistance in 2018–2019): Czechia, Portugal, and Slovenia; Group 3 (≥ 50% carbapenem resistance in 2018–2019): Bulgaria, Croatia, Cyprus, Greece, Hungary, Italy, Latvia, Lithuania, Poland, Romania, Slovakia and Spain.
